# Geostatistical Analysis of the Spatial Variation of *Chrysolina aeruginosa* Larvae at Different Stages in Desert Ecosystems

**DOI:** 10.3390/insects14040379

**Published:** 2023-04-12

**Authors:** Zeshuai He, Liangyue Chen, Ying Yang, Fuqiang Zhao, Chunmei Zhou, Dazhi Zhang

**Affiliations:** School of Life Science, Ningxia University, Yinchuan 750021, China; hezeshuai0614@stu.nxu.edu.cn (Z.H.);

**Keywords:** spatial distribution, *Chrysolina aeruginosa*, larvae, geostatistics, desert landscapes

## Abstract

**Simple Summary:**

The spatial distribution of pests is a prominent topic of research in population ecology. Knowledge of pest distribution patterns (including the specific plant segments that are most heavily infested) is of utmost significance in devising appropriate monitoring programs that will effectively reduce the utilization of pesticides. Geostatistical analysis, which reveals spatial correlations and dependencies, is vital in achieving an accurate comprehension of the disparities in pest spatial distribution. In this study, we utilized geostatistical methods to identify the spatial distribution of *Chrysolina aeruginosa*, a commonly occurring pest that attacks *Artemisia ordosica* in desert ecosystems of Ningxia, at various developmental stages. Our results indicated differences in the vertical distribution of larvae at distinct stages of growth. The number of larvae varies in relation to the height of the plants, and various morphological characteristics such as crown width and ground diameter. Furthermore, the larvae exhibit an inclination to aggregate.

**Abstract:**

*Chrysolina aeruginosa* is a major pest of *Artemisia ordosica*, and knowledge of the spatial distribution pattern of its larvae in their natural habitat is crucial for the implementation of effective control measures. This study employed geostatistical methods to investigate the damage caused by larvae of different age groups and their spatial distribution pattern. The distribution of *C. aeruginosa* larvae, which cause damage to *A. ordosica*, differed significantly according to their age. Younger larvae were predominantly found in the middle and upper parts of the plant, whereas older larvae were mainly distributed in the middle and lower parts, with significant differences in distribution location. A generalized linear model analysis revealed that the height of the plant, and plant morphological characteristics such as height, crown width, and ground diameter were significantly correlated with the number of larvae present. Furthermore, the interaction of age with other variables had an impact on the number of larvae. Kriging interpolation showed that *C. aeruginosa* larvae were distributed in aggregated patches with strong spatial heterogeneity. The younger larvae were more abundant in the center of the sample site, while the older larvae tended to be distributed toward the edges. These findings provide valuable information for designing effective control programs.

## 1. Introduction

The distribution of insect populations is determined by a combination of biological and environmental factors [1,2,3,4,5], which include the horizontal distribution (i.e., spatial distribution) and vertical distribution of individuals. The study of the distribution of individuals is crucial for sampling, investigating pest dynamics, determining control thresholds, and formulating effective control measures [6].

Spatial distribution patterns are typically described using traditional statistical methods such as binomial distribution, Taylor expansion analysis, and Iwao mean crowding regression. However, geostatistical methods can more accurately describe spatial correlations and dependencies. Ordinary kriging interpolation, in particular, can be used to predict values at unsampled locations in space, aiding in the prediction of pest spatial distributions and transmission dynamics [4,7,8,9,10].

The spatial distribution of insects is determined by the interplay of population dynamics with biotic and abiotic factors. The spatial distribution of pests is influenced by the morphological characteristics of plants, including plant height, crown width, and ground diameter [11,12,13,14,15]. First, higher plants can offer insects a larger habitat space and a greater food source, which may encourage the insects to congregate [14]. Second, a larger ground diameter of the plant is typically associated with more growth points and increasing nutrient availability, promoting healthy plant growth and reducing susceptibility to pest attacks. Conversely, plant parts with smaller ground diameters are weaker and more susceptible to pest infestations [16]. Crown width can also impact pest habitats and food sources, with wider canopies providing more favorable conditions for pest survival. Therefore, a comprehensive understanding of the factors influencing pest distribution can provide a theoretical foundation for the development of effective pest control strategies [17].

With regards to the vertical distribution of pests, many species prefer to feed on the upper parts of plants, which are closer to light and gas sources, and can provide optimal nutrients and water for pest growth and reproduction. However, some plants produce chemicals on their leaves that deter pest feeding, resulting in pests feeding on the lower portions of the plant [18]. During the spring and summer months, when temperatures are higher and plant growth is more robust, pests tend to inhabit the upper parts of plants, whereas during the autumn and winter months, when temperatures are cooler and plant growth is slower, pests tend to move to the lower parts of the plant [19,20]. Consequently, understanding the vertical distribution characteristics and clarifying the dynamic distribution law of pests can aid in the development of effective pest control strategies, which can reduce pest populations and improve plant quality.

*Artemisia ordosica* Krasch. (Asteraceae) is a drought-resistant plant with a lengthy growth period and strong sand-fixation capabilities [19,20]. It is widely cultivated throughout China and spans three natural zones, namely typical grassland, desertification grassland, and grassland desert [21,22]. This species plays a significant role in the restoration of desert vegetation [23].

*Chrysolina aeruginosa* (Faldermann, 1835) (Coleoptera, Chrysomeloidea) is a leaf-feeding pest that has rapidly spread in Northwest China in recent years causing significant damage to populations of *A. ordosica*, with 40–70% of damage reported in Inner Mongolia (Ningxia and Gansu provinces) [21,24,25]. Outbreaks of this pest have led to significant damage to the local ecology and economy, including the death of patches of *A. ordosica* and severe sanding of grassland [24,26]. According to Tian et al. [27], *C. aeruginosa* feeds solely on *A. ordosica*, and the maximum number of larvae in a single plant exceeds the number of adults. 

To date, only the spatial distribution of adult *C. aeruginosa* has been studied to some extent, while the larvae have been overlooked [28]. Research showed that during the larval developmental stage, the spatial distribution of larvae is influenced by a variety of factors, including larval behavior, host plant characteristics, and environmental conditions [4,5]. In this study, we aimed to study: (a) the vertical distribution of larvae; (b) the effect of plant morphology on larval distribution; and (c) the spatial distribution of larvae, providing insights into major damage sites and the spread of *C. aeruginosa* larvae of different ages. These findings may serve as a theoretical basis for future integrated pest management.

## 2. Materials and Methods

### 2.1. Study Area and Study Species

The research site was situated in Guyaozi within the National Nature Reserve of Lingwu Baijitan, Ningxia (between 106°38′21.79″ E–106°38′19.16″ E and 38°6′3.67″ N–38°6′17.27″ N, at an elevation of 1309–1319 m). The site covered approximately 6.4 ha, with a relatively even distribution of *A. ordosica* and good overall growth. *A. ordosica* accounted for 71.33% of the total vegetation cover, with a few other plant species including *Caragana korshinskii* Kom, *Sophora alopecuroides* L., *Suaeda salsa* (L.) Pall., *Salsola passerina* Bunge, *Oxytropis aciphylla* Ledeb., and *Cynanchum chinense* R.Br.

*C. aeruginosa* is a significant leaf-feeding pest of *A. ordosica* and undergoes four larval instars in one generation. The fourth instar larvae of *C. aeruginosa* overwinter in soil. The first and second instar larvae can consume up to half of the leaf, resulting in notched leaves, while the third and fourth instar larvae can completely defoliate the plant, and the adults feed on growing points and new leaves, ultimately causing the death of the entire plant [27]. Previous field studies indicated that *C. aeruginosa* larvae begin emerging in late September and start overwintering in late October. Therefore, three field surveys were conducted between 20 September and 18 October to investigate the population dynamics of *C. aeruginosa*. 

### 2.2. Experimental Procedures

**Figure 1:**
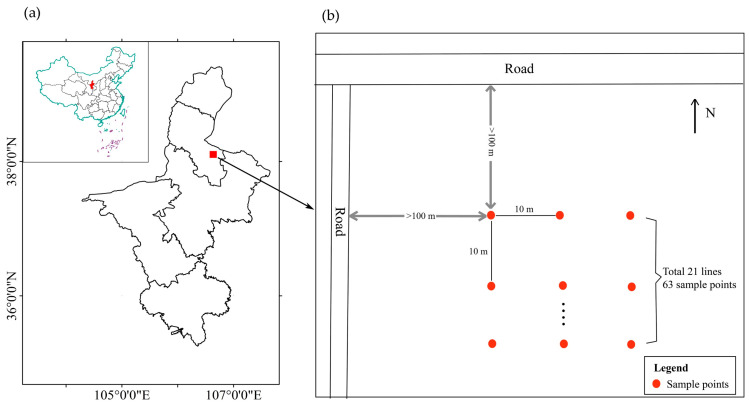
Schematic diagram of the plot. (**a**) Location map of the study area, (**b**) sampling design.

In the study area, a checkerboard sampling method was employed using 5 m × 5 m sample squares with a 10 m spacing between each sample point. A total of 63 sample points were set up for the study. To mitigate edge effects, sample points were located over 100 m away from the edge of the sample plots (as shown in Figure 1). For the study, one *A. ordosica* was chosen for each sample point, resulting in a total of 63 *A. ordosica* being surveyed three times (20 September, 4 October, and 18 October). During each survey, their geographical coordinates, plant height, crown width, and ground diameter were recorded.

#### 2.2.1. Population Survey of Different Instar Larvae

The number and age of all larvae present on each *A. ordosica* were recorded, alongside the geographic coordinates of *C. aeruginosa* larvae found on 63 *A. ordosica* trees. The larval stage was evaluated based on the criteria of morphology and size as outlined in Wei’s description of *C. aeruginosa* larvae [20]. Due to the similarity in morphology between the first and second instars, they are indistinguishable and were therefore aggregated for the purposes of statistical analysis [20,27]. 

#### 2.2.2. Vertical Distribution Survey of Different Instar Larvae

The larval count at various locations on each *A. ordosica* was recorded and subsequently categorized into five distinct height classes as follows: 0–20 cm, 21–40 cm, 41–60 cm, 61–80 cm, and 81–100 cm.

#### 2.2.3. Geostatistical Analysis

Spatially dependent patterns of *C. aeruginosa* larvae populations were analyzed using variance and ordinary kriging based on the larval count at each sample point [18]. Field observations were treated as a stochastic process denoted as *Z*(*x*), where *x* represents the spatial location. The semi-covariance, which is indicative of the spatial correlation between adjacent samples, was used to measure this correlation as follows:

(1) $$\gamma \left(h\right)=\frac{1}{2N(h)}\sum_{i=1}^{N(h)}{\left[Z\left(x_{i}\right)-Z\left(x_{i}+h\right)\right]}^{2}$$

where: *N*(*h*) is the number of measured pairs in the lag distance *h*; *Z*(*x_i_*) is the sample point measured at *x_i_*; *Z*(*x_i_* + *h*) is the sample point measured at *x_i_* + *h*. 

**Figure 2:**
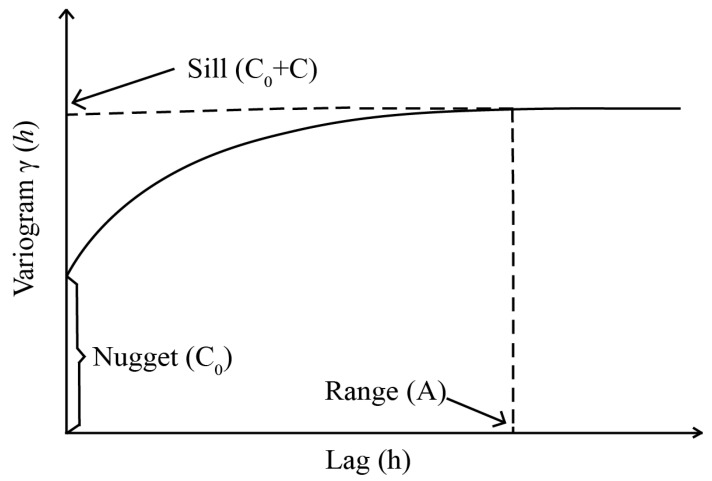
The variogram model illustrating nugget, sill, and range.

Figure 2 depicts the pattern diagram of the variance function, which consists of three ecologically significant parameters that influence the shape, structure, and ultimately, the spatial distribution of a population’s semi-covariance function. These parameters include the nugget (C_0_), the sill (C_0_ + C), and the range (A). The nugget represents the non-zero intercept point on the *y*-axis, indicating spatial variation due to sampling error and distance. The sill is the value at which the curve stabilizes on the *y*-axis, while the range denotes the average distance between points where spatial correlation exists [29,30].

As these estimates may fluctuate substantially from point to point due to sampling errors, models describing spatial variation must be fitted. We tested multiple models, including exponential, spherical, linear, and Gaussian models, using the following criteria to select the optimal one: an intercept (*β*_1_) near zero, a slope (*β*_0_) near 1, a large regression coefficient, a mean error close to zero, and a low root mean square error. Nugget effects, ranges, sill, and coefficients of determination were calculated for each model [4,31].

For the selected model, we calculated the level of spatial dependence (LSD) and determined the range values using the following equation:

(2) $$LSD=\frac{C_{0}}{C_{0}+C}$$

The nugget, denoted by C_0_, and the sill, represented by C_0_ + C, are used to describe the spatial autocorrelation of *C. aeruginosa* larvae. Additionally, strong, moderate, and weak spatial dependence are indicated by LSD values lower than 0.25, between 0.25 and 0.75, and higher than 0.75, respectively. The Geostatistical Analyst extension module in ArcGIS 10.4 (ESRI 2015) software was utilized to employ the kriging method for estimating values at unmeasured locations and creating predicted spatial distributions of *C. aeruginosa* larvae, as well as generating spatial distribution maps [32].

### 2.3. Statistical Analyses

To analyze the effect of plant morphological characteristics on the number of *C. aeruginosa*, a generalized linear model (GLM) with a Poisson distribution was used. We modelled the number of larvae according to their location at different sections of the plants (distribution height), plant height, crown width, and ground diameter. Additionally, the interaction terms of distribution height and plant height, crown width, and ground diameter of *A. ordosica* were included in the model. In order to prevent collinearity among the independent variables, according to the value of the variance inflation factor (VIF), the highly collinearity independent variables with VIF > 5 were eliminated. The data analysis was performed using SPSS^®^ 21. Plotting was performed using Origin^®^ 2023.

## 3. Results

### 3.1. Basic Information of C. aeruginosa Larvae

**Figure 3:**
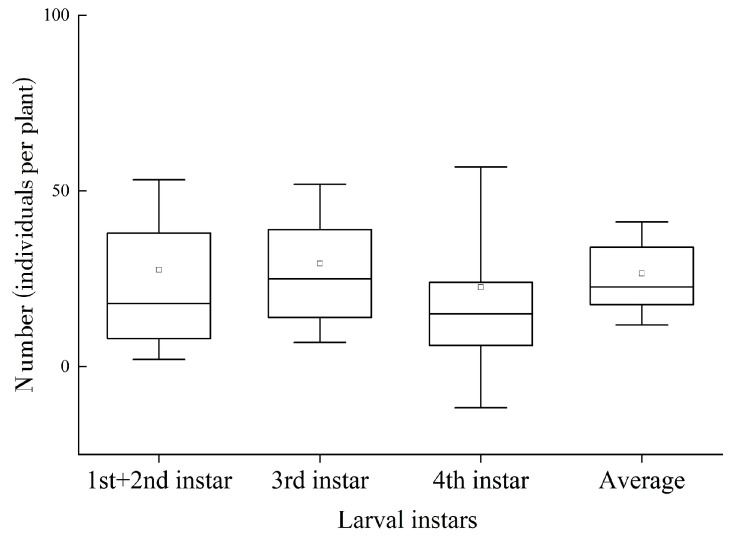
Number of specimens of *C. aeruginosa* larval instars (individuals per plant). Horizontal lines represent median value; circles represent average values; boxes represent upper and lower quartiles; vertical bars represent standard deviations.

A total of four larval instars were surveyed. The boxplots of the number of different ages are shown in Figure 3. Among the larvae of different instars, the third instar larvae had the largest number.

### 3.2. The Vertical Distribution of C. aeruginosa Larvae

**Figure 4:**
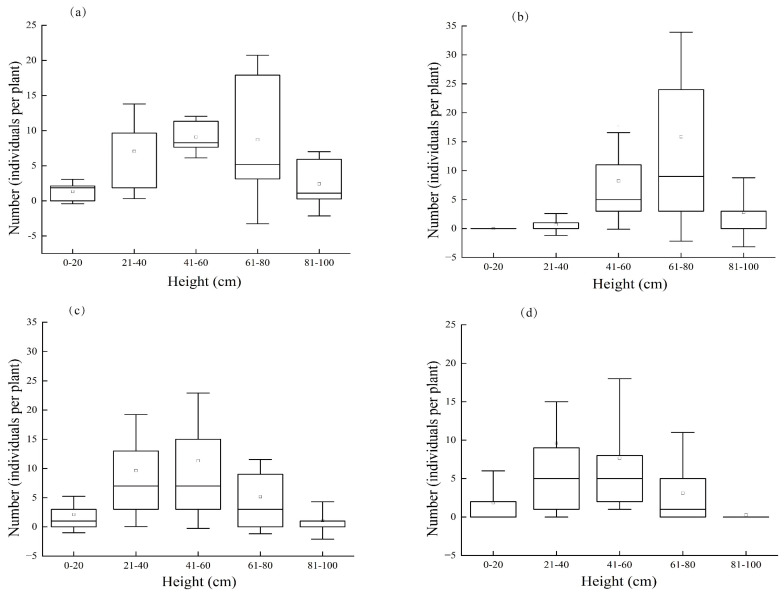
Vertical distribution of *C. aeruginosa* in different larvae instars. ((**a**) Vertical distribution of the mean number of larvae; (**b**) vertical distribution of first and second instar larvae; (**c**) vertical distribution of third instar larvae; (**d**) vertical distribution of fourth instar larvae). Horizontal lines represent median value; circles represent average values; boxes represent upper and lower quartiles; vertical bars represent standard deviations.

*C. aeruginosa* larvae had a clear vertical distribution on *A. ordosica*, with the upper–middle parts of *A. ordosica* being the most frequented (Figure 4a). The first and second instars were mainly distributed in the middle–upper parts (>60 cm) of the plant, the third instars were mainly found in the middle of *A. ordosica* (41–60 cm), while the older mature larvae were biased toward the middle–lower parts of *A. ordosica* (21–50 cm) (Figure 4b–d).

### 3.3. Correlation Analysis of the Number of C. aeruginosa Larvae and A. ordosica Characteristics

The variables contain classification variables; thus, the use of generalized linear models was deemed appropriate to analyze the relationship between variables. To reduce collinearity, independent variables with high variance inflation factor (VIF) were removed.

The GLM results revealed significant effects between the number of larvae on *A. ordosica* and distribution height, plant height, crown width, and ground diameter. Moreover, the number of larvae found on *A. ordosica* demonstrated a significant effect with the interaction between the position of the plant (distribution height) and ground diameter, as indicated in Table 1.

### 3.4. Geostatistical Analysis of C. aeruginosa Larvae

From the 12 semi-variogram models developed, four models were selected as optimal models (Table 2). Gaussian and spherical models were selected for different ages of larvae (Table 2 and Table 3).

**Figure 5:**
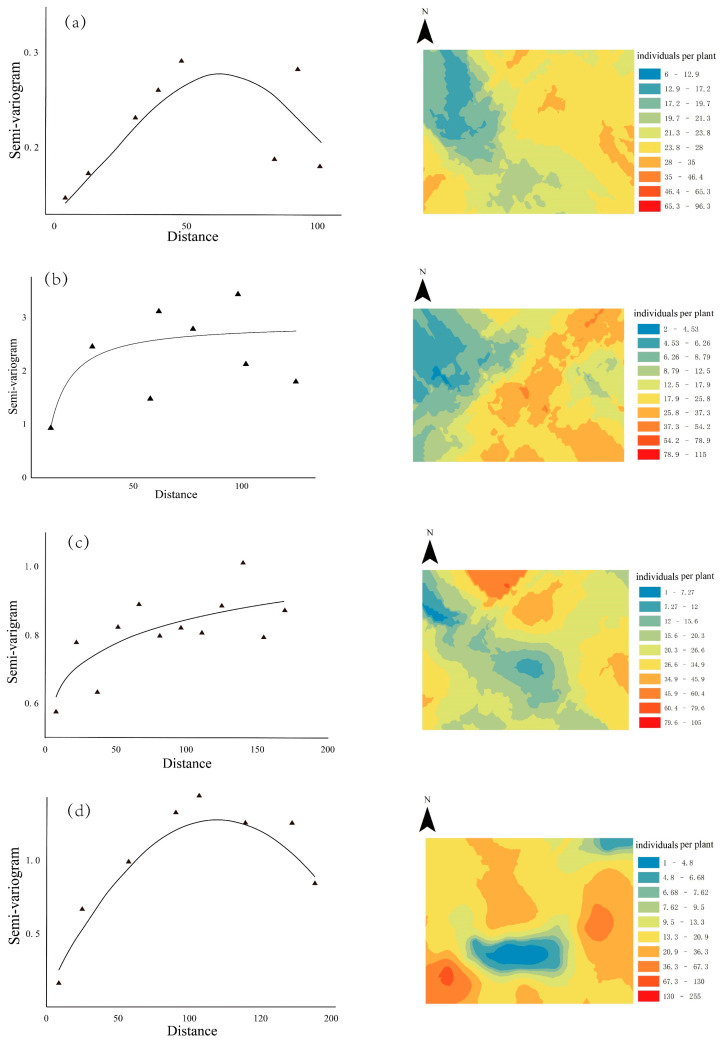
Semi-variogram curves (left panels) and kriging maps (right panels) of spatial patterns for *C. aeruginosa* larvae for the mean number of larvae (**a**), 1st and 2nd instar larvae (**b**), 3rd instar larvae (**c**), and 4th instar larvae (**d**).

Fitting parameters demonstrated that the larvae of *C. aeruginosa* exhibited patchy distribution patterns within the sampled plots (Table 3 and Figure 5). The edges of the plots had a higher frequency of occurrence compared to the center, and the spatial distribution displayed significant heterogeneity and aggregation. The nugget value was found to be 0.100, and the LSD value was 0.615, indicating a moderate level of spatial dependence and a small degree of randomness of the variables. Furthermore, the nugget value showed a gradual decrease, while the range increased with the larvae age, and the degree of spatial dependence shifted from strong to moderate over time.

The application of kriging for interpolating the abundance of *C. aeruginosa* larvae indicated that the spatial dependence varied across different larval age groups. Specifically, a higher concentration of young larvae was observed at the center of the sample plot compared to the edge, whereas as the larvae aged, the concentration at the edge surpassed that at the center.

## 4. Discussion

The examination of distribution patterns constitutes a significant component of research in population ecology, and it is vital in forecasting the dynamics of pest populations, enhancing sampling techniques, and formulating policies for pest management [30,33,34]. In this regard, this investigation employed geostatistical methods to investigate, for the first time, the spatial distribution of different larval instars of *C. aeruginosa* on *A. ordosica*.

Regarding vertical distribution, our study revealed that the first and second instars were predominantly concentrated in the upper–middle parts of *A. ordosica,* whereas the third instar larvae were mainly found in the middle section. These findings are consistent with the outcomes of prior studies by Tian et al., on the life history of *C. aeruginosa* [27]. Specifically, *C. aeruginosa* lays its eggs at the top of branches, enabling larvae to emerge effortlessly from their shells due to ample sunlight [20,27]. The hatchlings possess small mouthparts, rendering them incapable of feeding strongly, hence, making the upper shoots of branches easy targets. Conversely, as larvae mature into the fourth instar, they gradually descend downwards, gnawing on leaves for nourishment before eventually crawling into sand, where they overwinter as fully mature larvae [19,20,27].

By analyzing the variograms, it is possible to assess the degree of independence of infestations in nearby plants. A variogram that increases in value indicates a correlation between neighboring plants in the tested scales and directions [6]. In this study, the semi-variograms of the developmental stages of *C. aeruginosa* demonstrate that various larval instars possess small nuggets and large ranges. These results demonstrate that the spatial distribution of *C. aeruginosa* larvae is aggregated, indicating a spatial dependence of pest specimens over a spatial range. 

The degree to which insects exhibit aggregation is influenced by various factors, such as vegetation cover, species interactions, plant characteristics, pest abundance, anthropogenic disturbance, landscape composition, and geographic area [5,33,34,35,36,37,38,39,40,41]. Changes in vegetation cover in desert regions, which provides rich food resources and suitable habitats for desert insects, inevitably affect insect population patterns [42,43]. The spatial distribution of *C. aeruginosa* larvae is associated with the cover of *A. ordosica*, and its aggregation intensity increases with an increase in planting density. A high planting density of *A. ordosica* in a sample plot increases the aggregation distribution of larvae [28]. During field surveys, it was observed that the presence of boring pests in *A. ordosica*, such as *Holcocerus artemisiae*, *Adosomus* sp., and *Sphenoptera* sp., caused poor growth of *A. ordosica*, resulting in low egg production and uneven spatial distribution of *C. aeruginosa* larvae [21]. Different growth factors in *A. ordosica* can affect *C. aeruginosa* larvae differently. Tall plants with a high number of new shoots can provide a suitable environment for larvae to survive, allowing for a large accumulation of pests [44]. In this study, the GLM analysis revealed that the distribution height of pests and plant morphological characteristics (plant height, ground diameter, and crown width) had a significant impact on the number of larvae of *C. aeruginosa*. Additionally, the interaction term between larval distribution height and ground diameter also showed an impact on the number of larvae of *C. aeruginosa*. There were observed differences in the distribution of pests at different heights. Due to the height-oriented nature of *C. aeruginosa* larvae, the first and second instar larvae were found predominantly on the top of *A. ordosica*, moving gradually downward with development [20]. The taller the plant was, the more space there was available for insect distribution, and larger crown widths caused the larger feeding range of the larvae, ultimately influencing their spatial distribution. Conversely, the larger ground diameter of the plant led to a more scattered growth pattern [16], which was less conducive to the oviposition and larvae aggregation of *C. aeruginosa*. Interestingly, the interaction term between larval distribution height and ground diameter showed a positive effect, indicating that plants with larger ground diameters would attract more larvae at various distribution heights.

The spatial distribution of adults can impact the spatial distribution of larvae. Zhang et al.’s research on the spatial distribution of adults of *C. aeruginosa* indicates that adults are distributed in a non-random manner [28]. The findings of Zhang et al.’s study on the population dynamics of adults were consistent with the aforementioned research, with a higher number of adults observed in the upper region compared to the middle and lower regions [45]. After the adults lay eggs on the upper layer of the plant, the first and second instar larvae are primarily distributed in the middle and upper parts of the plant. As the larvae develop, they move downward, and the third and fourth instar larvae become mainly distributed in the middle and lower parts.

Anthropogenic disturbances can have a significant impact on the spatial distribution of species [46]. Blanchet et al. evaluated the reasons for the distribution of carabid beetles and found that human interference substantially affected the distribution of these insects [47]. In several different habitats, Zhang et al. found that human factors might be responsible for the differences in the distributions of adult *C. aeruginosa* [45]. Our survey revealed that grazing likely reduced the number of larvae around the sample plots, resulting in a heterogeneous spatial distribution. Investigating the influence of landscape structure on the spatial distribution patterns of pests in patches is important, as changes in landscape structure can affect the foraging behavior of phytophagous pests [48]. Further research is needed to determine how landscape changes can be regulated to reduce pest damage [23,49,50].

The edge effect is a common phenomenon in ecology, as pest abundance is generally greater at the edge of a sample than at its center [51,52]. Our results showed that the spatial distribution of *C. aeruginosa* larvae was consistent with that of most pests, with more individuals at the edge of the sample plot than in the center [4,9,53,54]. The main reason for the difference in the spatial distribution of the pests is the distribution of plants. Better plant growth leads to greater aggregation of pest individuals [2]. The quality and quantity of resources vary along the gradient from the patch edge to the interior, which affects the habitat quality for *C. aeruginosa* and subsequently leads to changes in the distribution of its larvae in response to *A. ordosica* growth conditions [2]. The center of the sample site is less populated than the edge, resulting in a higher rate of egg laying by adults in the center than at the edge. Since larvae do not migrate, the number of first and second instar larvae is greater in the center of the sample plot than at its edges. As the larvae mature, intra-specific competition increases, leading to a decrease in the population density of fourth instar larvae and a higher number of fourth instar larvae at the edge of the sample plot than at its center [19,55,56,57,58].

Integrated pest management includes chemical control, but the misuse of pesticides can lead to environmental pollution, pest resistance, and a significant decline in biodiversity [4]. Previous studies have shown that 50% caprylic acid or 2.5% deltamethrin insecticide preparations can effectively control *C. aeruginosa* [27]. By analyzing the spatial distribution of beetle larvae, pesticide applications can be planned according to their distribution, which optimizes the use of pesticides, reduces costs, and minimizes environmental pollution.

## 5. Conclusions

The infestation of *C. aeruginosa* has significant negative impacts on the growth of *A. ordosica* in China. In this study, we used geostatistical methods to determine the spatial distribution of different *C. aeruginosa* larval instars and also developed kriging interpolation models to map the distribution of *C. aeruginosa* larvae of different ages within the sample plots. At the same time, we investigated the vertical distribution of *C. aeruginosa* larvae on *A. ordosica*. Our findings provide a scientific basis for controlling *C. aeruginosa* infestations. For instance, starting treatment at the edges of *A. ordosica* sample plots may be more effective in reducing the pest’s population density. Additionally, information on the distribution of larvae at various ages can help concentrate control actions on the plant parts where larvae are more abundant. At a later stage, both ecological and chemical control methods should be used to minimize the damage caused by *C. aeruginosa* infestations.

## Figures and Tables

**Table 1:** Results of the generalized linear model (GLM) for the number of individual larvae of *C. aeruginosa* as a function of their location at different heights on the plants of *A. ordosica* and morphological characteristics (plant height, crown width, and ground diameter).

Variable	*df*	Chi-Square (χ^2^)	Estimate ± Standard Error	*p*
Intercept	1	152.847	1.124 ± 0.090	<0.0001
Distribution height	1	9.715	0.002 ± 0.001	0.0018
Plants height	1	12.647	0.004 ± 0.001	0.0004
Crown width	1	20.243	0.003 ± 0.001	<0.0001
Ground diameter	1	58.0876	−0.010 ± 0.001	<0.0001
Distribution height × Ground diameter	1	7.261	0.0001 ± 0.00002	0.007

*df* = degrees of freedom.

**Table 2:** Results of cross-validation of the semi-variogram models (spherical, exponential, and Gaussian) in the kriging method for *C. aeruginosa* different larval instars.

Age	Models	*β*_0_	*β*_1_	*R* ^2^	RMSE	ME
1st + 2nd instar larvae	Spherical	0.851	0.233	0.110	0.959	1.822
	Exponential	**0.880**	0.210	0.120	0.958	**1.819**
	**Gaussian**	0.874	**0.200**	**0.131**	**0.943**	1.867
3rd instar larvae	Spherical	1.143	0.072	0.025	0.929	1.569
	Exponential	1.194	0.089	0.020	0.908	**1.564**
	**Gaussian**	**1.113**	**0.044**	**0.030**	**0.902**	1.733
4th instar larvae	**Spherical**	**0.778**	0.050	**0.032**	**1.347**	**1.179**
	Exponential	0.777	**0.036**	0.021	1.357	1.194
	Gaussian	0.766	0.051	0.027	1.354	1.218
Average	**Spherical**	**1.052**	**−0.001**	**0.013**	**0.612**	**0.582**
	Exponential	1.052	−0.001	0.013	0.613	0.598
	Gaussian	1.057	0.005	0.005	0.596	1.023

*β*_0_ and *β*_1_ are the slope and intercept of the kriging cross-validation curve, respectively. RMSE = root mean square error, ME = mean error, and *R*^2^ = coefficient of determination from the cross-validation curve. Model selection was based on the following criteria: lowest root mean square error (RMSE), highest coefficient of determination (*R*^2^), slopes (*β*_0_) and intercepts (*β*_1_) of the cross-validation close to one and to zero, respectively, and ME close to zero. Selected models are in bold.

**Table 3:** Model parameters and spatial patterns of *C. aeruginosa* larvae.

Age	Model	Range	*C*_0_	*C* + *C*_0_	LSD	Distribution
1st, 2nd instar larvae	Gaussian	51.772	0.932	1.031	0.096	Aggregate distribution
3rd instar larvae	Gaussian	117.952	0.670	0.868	0.228	Aggregate distribution
4th instar larvae	Spherical	131.278	0.365	1.178	0.690	Aggregate distribution
Average	Spherical	52.500	0.100	0.260	0.615	Aggregate distribution

*C*_0_ = nugget effect; *C*_0_ + *C* = sill; LSD = *C*/(*C*_0_ + *C*) = level of spatial structuration.

## Data Availability

The data presented in this study are available on request from the corresponding author.
